# Decoding the complexities of lipid homeostasis through a unified mouse genetic resource

**DOI:** 10.1016/j.jlr.2024.100511

**Published:** 2024-01-28

**Authors:** Carlos Viesi, Marcus Seldin

**Affiliations:** 1 Department of Biological Chemistry, University of California, Irvine, USA; 2 Center for Epigenetics and Metabolism, University of California, Irvine, USA

**Keywords:** lipoprotein, GWAS, QTL, diversity outbred, mouse genetics

Lipoproteins are particles that transport fats (lipids) through the bloodstream. They differ in size and density based on the amount of lipids and proteins they carry. They are broadly broken down into several categories: Chylomicrons, which are the largest and least dense lipoproteins, transport dietary triglycerides and cholesterol from the intestines to the liver and other tissues. VLDLs are larger particles that carry triglycerides synthesized in the liver to tissues. As triglycerides are removed, VLDL particles become smaller and denser. Intermediate-density lipoproteins (IDLs) are formed from the breakdown of VLDL as triglycerides are removed. LDLs are smaller and denser than VLDL and IDL. They mainly transport cholesterol to tissues, earning them the label "bad cholesterol" when levels are high as they can contribute to plaque buildup in arteries. HDL, the smallest and densest lipoproteins, collect excess cholesterol from tissues and transport it back to the liver for disposal or recycling, earning them the title "good cholesterol." The size and density differences relate to their function in lipid transport. Larger particles like chylomicrons and VLDL primarily carry triglycerides, while smaller and denser particles like LDL and HDL primarily carry cholesterol, although the contents can shift based on conditions such as diet and/or genetics. High levels of certain lipoproteins, particularly LDL, can increase the risk of CVD as they contribute to plaque formation in arteries, while higher levels of HDL are associated with a reduced risk due to their role in cholesterol clearance. Genetic analyses such as association mapping have been central in relating the complex nature of circulating lipoproteins to metabolic diseases. For example, genome-wide associations in large-scale patient cohorts have prioritized key enzymes and pathways linking plasma lipids to clinical phenotypes (1, 2, 3). These associations have led to the prioritization of actionable treatment strategies in CVDs, such as prioritization of Patatin-like phospholipase domain–containing protein 3 in a spectrum of liver diseases related to its role in lipid metabolism (4, 5). In addition, post hoc analyses of large-scale lipoprotein genome-wide association studies have guided causality inference for disease mechanisms. For example, mendelian randomization analyses following generation of these cohorts have prioritized a causal role for LDLs in driving CVD (6). While large clinical cohort studies have reaffirmed epidemiologic correlations between various lipoprotein abundances and cardiometabolic outcomes, defining the nature of their synthesis, secretion, transport, and metabolism remain a significant challenge.

Mouse models have been critical in guiding our understanding of lipid homeostasis and complex diseases, given their ease of genetic manipulation, decades of experimentation built on fixed genotypic backgrounds exposed to diverse environmental conditions, as well as accessible nature to many researchers (7). The use of genetic reference panels has enabled bridging a wealth of physiologic studies in mouse models with natural genetic diversity (8). In a recent study in the *Journal of Lipid Research*, Price and colleagues generated a new resource to explore interactions between lipoprotein size and genetic variation (9). Initially, they examined lipid particle size in normal chow and Western diets of both sexes of all eight parental strains, which comprise the diversity outbred population. These measurements allowed the authors to estimate the genetic contribution (or heritability) to variation in particle sizes, which was ∼60% under normal chow conditions but reduced in the cohort fed a Western diet. Following the intuition that genetic background is a significant contributor to regulation of lipoprotein size, and expanded the study to assay 16-plasma lipoprotein particles in over 500 Diversity Outbred (10) mice on a Western diet. The large sample size enabled the authors to apply association mapping and identify 21 quantitative trait loci (QTLs), where 18 gene orthologs corresponded to conserved human lipid associations. The effects of several notable QTLs could be traced back to parental strains, which contributed more allelic variations to these outcomes, such as 129 genotypes driving a hotspot on chromosome 1 and CAST on chromosome 9. To provide mechanistic validation for this new resource, the authors selected a locus association to multiple lipid species, where N-acyl sphingosine amidohydrolase 2 (*Asah2*), a ceramide-catabolizing neutral ceramidase (11) resided. They observed that *Asah2* is a master regulator of circulating lipids in that levels of HDL, midzone, large LDL, IDL, and small VLDL were significantly altered in the plasma of mice comparing KO to litter-match controls. The effects of *Asah2* mutation also showed a clear sex-specific pattern in that the KO showed a stronger effect in females than males. Clearly, in vivo, manipulation of candidate genes from association mapping outcomes similar to *Asah2* will be instrumental in deducing causal pathways in lipid metabolism and disease. To suggest conserved mechanisms of human cardiometabolic traits linked to lipoprotein sizes, the authors conclude by aggregating syntenic region associations in mice to human genome-wide associations to relevant outcomes such as glycemic traits and CVDs.

Beyond extending our knowledge of actionable mechanisms driving systemic lipid regulation, this new resource presents additional appeal in that the data builds on a wealth of comprehensive phenotypic and molecular characterizations performed on the same Diversity Outbred mice. Similar to other mouse genetic reference panels such as the C57BL6/J × DBA/2J (BXD) (8, 12) and Hybrid Mouse Diversity Panel (13), tight environmental control and a wide range of measures enables researchers to integrate across data scales to dissect complex physiologic traits. Thus, genetically driven pathways identified through associations to lipid sizes could be further characterized in an unbiased fashion by integrating observations with previous QTLs and correlations listed in Table 1. One such example would be to intersect loci which comap to both lipoprotein size and islet functional parameters (14) to inform potential conserved mechanisms of lipid and glycemic homeostasis. As more data such as RNA-sequencing, metabolite quantifications, or proteomics are layered on top of these data, a deeper understanding of genetically varied mechanisms regulating and responding to lipoprotein size will be achievable. Future applications of additional informatics-based methods such as network modeling and/or machine learning to characterize physiologic outcomes within these integrated datasets will also enable a deeper understanding of lipid homeostasis and relationships to disease.

**Table 1:** Summary of DO screens using matched cohort to current study

Study	Number of DO Mice	Summary	Reference (PMID)
Price *et al.*	500	Lipoprotein size quantification	37944753
Price *et al.*	384	Liver multiple phospholipids	37523383
Zhang *et al.*	264	Microbial abundances, intestinal lipids and host traits	36759753
Keller *et al.*	483	Ex vivo pancreatic islet phenotypic screening paired with host metabolic traits (https://churchilllab.jax.org/qtlviewer/attie/islets)	31343992
Linke *et al.*	385	Plasma and liver mass-spec lipidomic quantification (http://www.lipidgenie.com/qtlViewer.html)	32958938

DO, Diversity Outbred.

## Conflict of interest

The authors declare that they have no conflicts of interest with the contents of this article.
